# Adrenal Metastases as Sanctuary Sites in Advanced Renal Cancer

**DOI:** 10.15586/jkcvhl.2020.132

**Published:** 2020-10-12

**Authors:** Ulka Vaishampayan, Harsh Shah, Mohammad F. Asad, Dongping Shi, Brenda Dickow, Stacey Suisham, Jason Domina, Michael L. Cher, Julie Samantray, Hussein D. Aoun

**Affiliations:** 1 Department of Oncology, Karmnos Cancer Center/Wayne State University, Detroit MI, USA;; 2 Department of Pathology, Karmanos Cancer Institute, Wayne State University, Detroit, MI, USA;; 3 Department of Radiology, Karmanos Cancer Institute, Wayne State University, Detroit, MI, USA;; 4 Department of Urology, Karmanos Cancer Center/Wayne State University, Detroit, MI, USA;; 5 Division of Endocrinology, Department of Medicine, Karmanos Cancer Center, Wayne State University, Detroit, MI, USA

**Keywords:** kidney cancer, immunotherapy, clear cell, metastases

## Abstract

Involvement of the adrenal gland in kidney cancer represents a unique site of metastasis with a distinct clinical course. The cases are typically resistant to immune therapy and need local therapy management. A case series of patients with adrenal metastases was reviewed to highlight the nuances of clinical course and therapy. We reviewed renal cancer carcinoma (RCC) cases with adrenal metastases at Karmanos Cancer Center, Detroit MI. Medical records were reviewed to collect relevant case information. Next-generation sequencing, tumor mutation burden testing, and programmed death ligand biomarkers were evaluated in five cases. Twelve cases were reviewed; all were males with a median age of 49.5 years. Three patients presented with adrenal metastases only and were treated with local therapy. Three received interleukin-2 (IL-2). One patient relapsed with bilateral adrenal lesions after 11 years of remission, post-IL-2 therapy. Five cases received immune checkpoint inhibitor (ICI) and one received antivascular therapy. ICI therapy was followed by ablation of residual adrenal metastases in three patients. Genomic profiling was available in five cases. All were *BAP1* and PD-L1 negative.Pathogenic mutations in *PBRM1, SETD2*, and *VHL* were noted. All patients with residual adrenal metastases responded to antivascular therapies or to local ablation. One patient died 17 years after diagnosis and 11 patients are alive at a median follow-up of 9.5 years. Adrenal metastases in RCC have a distinct clinical course. They can represent a sanctuary site of relapse/residual disease following treatment with immune therapy. Management with local therapy can induce durable remissions. Systemic management with antivascular therapies also demonstrated favorable responses. Further investigation should focus on the unique clinical course and optimal management of adrenal metastases in kidney cancer.

## Introduction

Renal cell carcinoma (RCC) is a heterogenous disease. The spectrum of clinical outcomes can range from overall survival of few months to many years. The current risk profiling established by the International Metastatic Disease Consortium (IMDC) involves the use of clinical factors such as time from nephrectomy, anemia, performance status, calcium, lactate dehydrogenase levels, platelet count, and neutrophil count (1). The IMDC criteria prognosticate patients into favorable (0 factors), intermediate (1–2 risk factors), and poor risk (3 or more factors) categories. Despite these standardized criteria, there are multiple other factors that determine clinical outcome and require individualized management. Sites of metastases in RCC have been noted as an important prognostic factor. For example, patients with lungs only metastases were shown to have a better outcome than the patients with other sites of metastases (2). Pancreas metastases

in RCC have been reported to demonstrate an especially prolonged disease course (3, 4). Patients with bone metastases have worse outcomes (5). Adrenal metastasis as a special category with unique clinical outcomes has not been reported so far within kidney cancer.

Immune checkpoint therapies (ICI) have changed the landscape of kidney cancer therapeutics (6). The potential for durable remission is an attractive feature of ICI-based therapy. A favorable toxicity profile than the cytokine regimens has enabled wider clinical applications of ICI therapies. We observed multiple cases that had adrenal metastases as the only residual sites, or sites of relapse after immune-based regimens. Our clinical observations noted a distinct clinical course in RCC patients with adrenal metastases. A unique pattern of resistance to ICI was also noted in RCC cases with adrenal metastases. We compiled a case series of RCC patients with adrenal metastases to evaluate the features of clinical course, presentation, and response to ICI therapy. The report also sheds light on the nuances of specific clinical management of adrenal metastases cases within RCC.

## Patients and Methods

The protocol was reviewed and exemption was granted by the Wayne State University Institutional Review Board. We reviewed a case series of patients at Karmanos Cancer Center with advanced RCC who presented with adrenal metastases from RCC, either as a solitary site or as dominant areas of relapse/residual disease. Medical records were reviewed to collect patient information regarding demographics, sites of metastases, IMDC prognostic risk characteristics, therapy, response rates, and progression free and overall survival. Molecular profiling data were collected if available.

### Tissue testing

#### Next-generation sequencing

Next-generation sequencing (NGS) was performed on genomic DNA isolated from formalin-fixed paraffin-embedded (FFPE) tumor samples using the NextSeq platform (Illumina Inc., San Diego, CA, USA). A custom-designed SureSelect XT assay (Agilent Technologies, Santa Clara, CA, USA) was used to enrich the 592 whole-gene targets that comprised a 592-gene NGS panel. All variants were detected with >99% confidence based on allele frequency and baited capture pull-down coverage with an average sequencing depth of over 500X and an analytic sensitivity of 5% variant frequency.

#### Total mutation burden

Total mutation burden (TMB) was calculated based on the number of nonsynonymous somatic mutations identified by NGS while excluding any known single nucleotide polymorphism (SNP) in dbSNP (version 137) or in the 1000 Genomes Project database (phase 3; http://www.internationalgenome.org/). TMB is reported as mutations per megabase sequenced. The threshold for determining high TMB as greater than or equal to 10 mutations/megabase was established.

#### PD-L1 immunohistochemistry

PD-L1 immunohistochemistry (IHC) analysis was performed on slides of formalin-fixed paraffin-embedded (FFPE) tumor samples using automated staining techniques. The procedures met the standards and requirements of the College of American Pathologists. The primary antibody against PD-L1 was SP142 (Spring Bioscience, Pleasanton, CA, USA), except for Non-small cell lung cancer (NSCLC) tumors tested after January 2016. For NSCLC tumors tested after January 2016, the primary PD-L1 antibody clone was 22c3 (Dako, Santa Clara, CA, USA). For the calculations in this manuscript, staining for both antibodies was considered positive if there was staining on ≥1% of tumor cells.

## Results

### Clinical results

**Figure 1:**
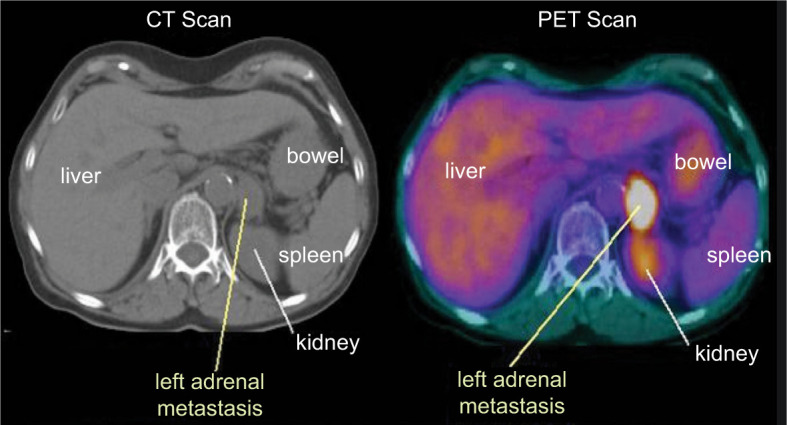
Computed tomography and positron emission tomography imaging showing adrenal gland metastasis.

**Figure 2:**
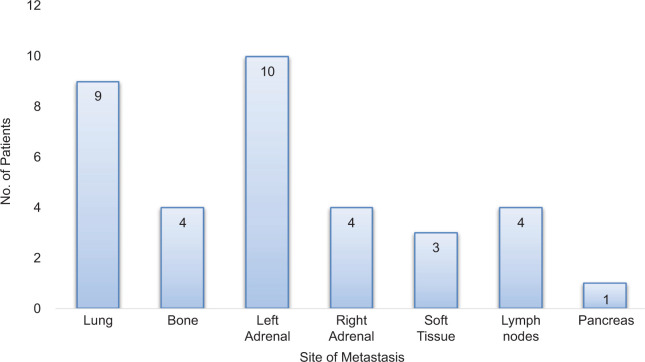
Distribution of location of distant metastases in nine patients with multiple sites of metastases.

Twelve patients were included in this retrospective report. Eleven had clear cell histology, but one had translocation-type histology. Patient characteristics are summarized in Table 1. The median time from RCC diagnosis to adrenal metastasis was 68 months (range 0–252 months), and the median time from initial metastatic disease to appearance of adrenal metastasis was 15 months (range 0–111 months). A typical scan showing adrenal metastases is depicted in Figure 1. The sites of metastases are summarized in Figure 2. Four patients had bilateral adrenal metastases.

**Table 1:** Case summaries of advanced renal cancer with adrenal metastases.

Patient characteristic	No. (%)
Median age (range)	49.5 years; range 41–80 years
Gender: male/female	12 (100%)/0 (0%)
Race: AA/CA/Hispanic	1 (8.3%)/10 (83.4%)/1 (8.3%)
Unilateral/bilateral	9 (75%)/3 (25%)
Nephrectomy	11 (91.7%)/1 (8.3%)
Histology: clear cell/translocation xp11	11 (91.7%)/1 (8.3%)
Fuhrman Grade: 2/3/unknown	5 (42%)/4 (33%)/3 (25%)
Median time to adrenal mets (range)	68 months (0–252 months)
Systemic therapy: IL-2/ICI/none/VEGF	3 (25%)/5 (41%)/3 (25%)/1 (9%)
Systemic therapy: anti-VEGF therapy	6 (50%)
Local therapy: Surgery/cryotherapy/microwave/none	3 (25%)/6 (50%)/1 (8%)/2 (17%)
IMDC risk: Fav/Int/poor	4 (33.3%)/8 (66.7%)/0 (0%)

AA, African American; CA, Caucasian; IMDC, International Metastatic Disease Consortium; Fav, favorable; Int, intermediate; IL-2, interleukin-2; ICI, immune checkpoint inhibitor; mets, metastases; VEGF, vascular endothelial growth factor.

**Figure 3:**
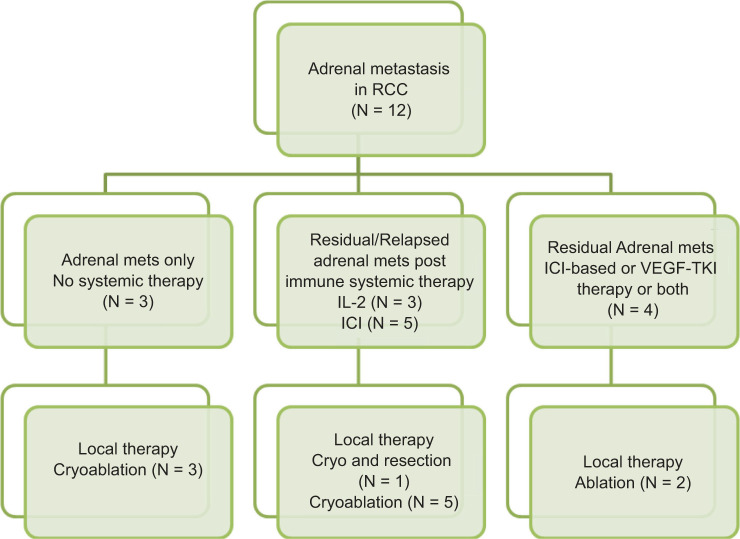
Management flow chart for adrenal metastases in renal cancer. Overlap is seen as some patients received multiple different types of therapies.

Three patients presented with adrenal metastases only, and were treated with local therapy and achieved complete remission. Three received high-dose IL-2. One patient received high-dose IL-2 for bone metastases and relapsed with a solitary adrenal metastasis 11 years later, which was treated with cryotherapy. The same patient developed metastasis in the other adrenal 18 months later, which was treated with surgical resection. He was rendered into complete remission after surgical resection and continued with no evidence of disease 1 year later. Of the other two patients receiving IL-2 therapy, one had unresectable large adrenal masses and is now demonstrating response to antivascular therapy. The other patient progressed on IL-2 but demonstrated a response to ipilimumab and nivolumab at all sites except in the adrenal gland. The adrenal lesion was treated with cryoablation. Five cases received immune checkpoint inhibitor (ICI) and one received antivascular therapy. ICI-based regimens were ipilimumab + nivolumab in two patients, and axitinib and pembrolizumab, a combination of avelumab and an adenosine receptor inhibitor and bevacizumab and atezolizumab, each in one patient. ICI therapy was followed by ablation of residual adrenal metastases in three patients. All patients with residual adrenal metastases post-immune therapy responded to antivascular therapies or to local ablation. With a median follow-up of 9.5 years (range: 4 years to 26 years), one patient had expired 17 years after diagnosis. Eleven patients are alive and follow-up is ongoing. The systemic and local therapy management is summarized in Figure 3.

### Genomic Results

Next-generation sequencing and PD-L1 testing results are available on five cases, which are summarized in Table 2. All cases were PD-L1 negative by IHC. None of the cases had *BAP1* mutations. Pathogenic mutations in *PBRM1, SETD2*, and *VHL* were noted. Five patients, all with clear cell histology, had genomic testing done, of which three were on adrenal metastasis tissue. Three cases had *PBRM1* pathogenic mutations, of which two also had *SETD2* pathogenic mutations. Four cases demonstrated pathogenic *VHL* mutations. Tumor mutation burden was low at <5 mutations/megabase in all cases. None of the patients had *BRCA1* or *BRCA2* mutations or any other DNA repair mutations. One case with *ATM* mutation was deemed a variant of unknown significance. Two cases had NGS conducted on both primary and metastases (adrenal and mediastinal metastasis). No major differences were noted between NGS results from primary tumor tissue and metastatic biopsies.

**Table 2:** Clinical course and molecular profile of cases.

Case number/location	NGS/IHC results	Clinical course
Case 1 Adrenal	*PBRM1* exon 21 *KDM5C* Exon 4 TUBB3 40% ERCC10%	Received IL-2 for RCC with bone mets with CR Relapsed with bilateral adrenal metastases, 11 and 12.5 years after IL-2 therapy. Treated with local therapy, cryo therapy on one lesion and surgery on the other.
Case 2 KidneyAdrenal	*PBRM1 exon 18* *SETD2* exon 10 *VHL* exon 1 *PBRM1* exon 18 *SETD2* exon 10 *VHL* exon 1	Clear cell RCC post-nephrectomy Relapsed with adrenal metastasis, treated with local therapy. No systemic therapy.
Case 4 KidneyMediastinal	*VHL* exon 1 *CDKN1B* exon 1 *VHL* exon 1 *CDKN2A* exon 2	Clear cell RCC post-nephrectomy Relapsed with lung metastases 46 months after and treated with ICI.Response seen in lung, but adrenal metastases appeared 67 months after nephrectomy as sites of PD during ICI therapy. Responding well currently to TKI therapy.
Case 6 Kidney	*SETD2* exon 3 *ATM* VUS exon 26 *VHL* exon 2	Synchronous presentation with kidney mass and metastases to lung and bone. Received ICI therapy and had response at other sites but PD with new adrenal metastases. Responding well currently to TKI therapy.
Case 7 AdrenalLung	*PBRM1* exon 26 *SETD2* exon 16 *VHL* exon 2 ERCC 100% *IHC*	Post-nephrectomy presented with lung metastases. Received IL-2 and progressed. Treated with ICI and had a response in lung but adrenal metastasis emerged. Treated with local therapy.

NGS, next-generation sequencing; IHC, immunohistochemistry; PD-L1, programmed death ligand-1; Int, intermediate; VUS, variant of uncertain significance; IL-2, interleukin-2; ICI, immune checkpoint inhibitor; RCC, reviewed renal cancer.

## Discussion

Immune checkpoint inhibitor-based regimens in RCC have improved clinical outcomes and higher response rates than those seen previously with single agent tyrosine kinase inhibitor (TKI) (6, 7). We report here a specific organ site of metastasis, such as the adrenal gland demonstrating resistance to immune therapy. Management with either local therapy alone or a combination of systemic therapy with TKI such as pazopanib or cabozantinib followed by local therapy is required. The presentation of adrenal metastases in RCC falls into the following three main categories: (i) De novo presentation with adrenal only metastases; (ii) residual/sanctuary adrenal metastases that are the only sites of disease resistant to immunotherapy; and (iii) adrenal metastases as a component of overall disease progression, in which case novel systemic therapies need to be explored.

Local therapy management for adrenal metastases consists of surgical resection, minimally invasive cryoablation/microwave ablation, or stereotactic radiation therapy. Systemic therapy with vascular endothelial growth factor tyrosine kinase inhibitor (VEGF-TKI) should be considered to achieve tumor size reduction and then attempt local therapy to render complete remission. The recent results of combinations of stereotactic body radiation therapy (SBRT) and immune checkpoint-based systemic therapy studies have demonstrated response rates and overall outcomes no different than those with systemic therapy alone (8, 9). However, if adrenal lesions are present as a sanctuary site after immune therapy, then use of local therapy is indicated. Successful and safe local therapy of adrenal metastases with surgical resection, percutaneous cryoablation/microwave ablation, and SBRT, has been established (10–13). A series of melanoma cases presenting with solitary adrenal metastases that were treated with local therapy has been reported. No systemic therapy was utilized in those cases (14). A recently reported series consisted of two melanoma cases, and one uterine carcinosarcoma case, presenting with adrenal residual metastasis after pembrolizumab (15). Local therapy was effective in treating adrenal lesions in these cases. The prolonged follow-up in our case series shows that in RCC these cases are anticipated to have long-term remissions, especially as the other metastatic sites demonstrated durable remission after ICI therapy.

Molecular profiling enables delivery of personalized therapy and is gradually gaining importance in therapeutic decisions in malignancies. In RCC, the clinical application of NGS biomarkers has not been established. The lack of *BAP1* mutations in these tumors is consistent with the indolent disease course and better prognosis (16). The lack of programmed death-ligand 1 (PD-L1) expression and low TMB suggest resistance to ICI therapy. *PBRM1* loss of function mutations appear to be predictive of higher likelihood of response to ICI therapy (17). *VHL* mutations were consistent with a response to therapies targeting VEGF. The resistance to ICI therapies is not explained on the basis of known biomarkers from contemporary testing. Exploring other mechanisms of resistance by evaluating proteomics, epigenomics, or metabolomics maybe required (18).

The reasons for adrenal gland lesions presenting as sanctuary sites are not clear. The simplistic explanation appears to be that the tumor microenvironment of glucocorticoids within adrenal cortex maybe responsible for development of immune-resistant metastases (19). The immunosuppressive milieu is likely to prevent tumor infiltration of lymphocytes (TIL), hence promoting progression of metastases (20). Inflammatory biomarkers and presence of TIL may indicate pathways of resistance to immune checkpoint inhibition and are worthy of the future investigation. The limitations of our study are the small sample size and the retrospective design. However, the findings of a consistent clinical course with prolonged follow-up, generate the hypothesis of a unique subgroup within RCC. The findings need to be validated in a larger multicenter trial.

## Conclusion

Adrenal metastases were noted to be a distinct pattern of relapse or sanctuary site in advanced RCC. The relapse/residual disease in adrenals was noted despite durable remission at other sites. Local management was effective and systemic therapy with anti-VEGF therapies demonstrated response. No association with currently known genetic mutations was noted. The immunosuppressive tumor environment milieu in adrenal cortex maybe potentially responsible for adrenal sites of metastases that are resistant to immune therapy. Further investigation should focus on this unique pattern of relapse and optimal management.
